# Supplementary material for: Booster immunization of meningococcal meningitis vaccine among children in Hangzhou, China, 2014-2019
Source: PLoS One. 2021 May 25;16(5):e0251567. doi: 10.1371/journal.pone.0251567 (PMC8148366; doi:10.1371/journal.pone.0251567)
Supplement: S1 Table — Since the 1980s, the number and incidence of meningitis in Hangzhou have been gradually decreasing. The periodic prevalence of meningitis has disappeared in Hangzhou and has been sporadic for many years, with zero incidence in some years. In recent years, both urban and suburban areas are in a sporadic state, and there is no cluster epidemic. (DOCX) [file pone.0251567.s001.docx]

**S1 Table. Month distribution of meningococcal meningitis in Hangzhou from 1950 to 2019 (No. of cases).** Since the 1980s, the number and incidence of meningitis in Hangzhou have been gradually decreasing. The periodic prevalence of meningitis has disappeared in Hangzhou and has been sporadic for many years, with zero incidence in some years. In recent years, both urban and suburban areas are in a sporadic state, and there is no cluster epidemic.

| Age | 1976-1979 | 1980-1989 | 1990-1999 | 2000-2009 | 2010-2019 | Total |
| --- | --- | --- | --- | --- | --- | --- |
| 0- | 164 | 151 | 7 | 7 | 2 | 331 |
| 1- | 57 | 55 | 5 |  |  | 117 |
| 2- | 99 | 44 | 7 | 3 |  | 153 |
| 3- | 163 | 57 | 5 | 3 |  | 228 |
| 4- | 164 | 66 | 3 | 3 |  | 236 |
| 5- | 193 | 80 | 7 | 1 |  | 281 |
| 6- | 177 | 82 | 8 |  |  | 267 |
| 7- | 217 | 83 | 7 | 1 |  | 308 |
| 8- | 223 | 72 | 6 | 2 |  | 303 |
| 9- | 253 | 48 | 8 | 3 |  | 312 |
| 10- | 1031 | 268 | 29 | 10 |  | 1338 |
| 15- | 925 | 330 | 9 | 4 |  | 1268 |
| 20- | 589 | 371 | 14 | 3 | 4 | 981 |
| 30- | 194 | 125 | 8 |  |  | 327 |
| 40- | 110 | 72 | 7 | 2 | 2 | 193 |
| 50- | 107 | 54 | 2 | 1 | 1 | 165 |
| 60以上 | 59 | 40 | 1 | 3 |  | 103 |
| unknown | 59 | 11 |  |  |  | 70 |
| Total | 4784 | 2009 | 133 | 46 | 9 | 6981 |
