# Supplementary material for: Booster immunization of meningococcal meningitis vaccine among children in Hangzhou, China, 2014-2019
Source: PLoS One. 2021 May 25;16(5):e0251567. doi: 10.1371/journal.pone.0251567 (PMC8148366; doi:10.1371/journal.pone.0251567)
Supplement: S2 Table — Another study in Hangzhou showed that the antibody concentration of group A or group C in the subjects would increase with increasing immunization times, and the average serum antibody concentration was the highest in people who received 4 doses of meningococcal vaccine. It can be seen that the two doses of basic immunization within 18 months of age and one dose of booster immunization at 3 and 6 years of age can produce high concentration of antibodies. (DOCX) [file pone.0251567.s002.docx]

**S2 Table. Nm^a^ antibody levels in group A and group C of healthy people in an urban area of Hangzhou in 2018.** Another study in Hangzhou showed that the antibody concentration of group A or group C in the subjects would increase with increasing immunization times, and the average serum antibody concentration was the highest in people who received 4 doses of meningococcal vaccine. It can be seen that the two doses of basic immunization within 18 months of age and one dose of booster immunization at 3 and 6 years of age can produce high concentration of antibodies.

| No. of immune | serum antibody of group A | | | | serum antibody of group C | | | |
| --- | --- | --- | --- | --- | --- | --- | --- | --- |
|  | No. of survey | No. of protected | The antibody protection rates (%) | GMC^b^  (μg/mL) | No. of survey | No. of protected | The antibody protection rates (%) | GMC^c^  (μg/mL) |
| 0 | 145 | 120 | 82.76 | 8.14 | 154 | 47 | 30.52 | 2.38 |
| 1 | 7 | 6 | 85.71 | 9.48 | 18 | 16 | 88.89 | 8.78 |
| 2 | 47 | 34 | 72.34 | 5.75 | 81 | 69 | 85.19 | 10.56 |
| 3 | 40 | 38 | 95.00 | 11.46 | 32 | 30 | 93.75 | 14.75 |
| 4 | 60 | 58 | 96.67 | 16.69 | 14 | 14 | 100.00 | 15.35 |
| total | 299 | 256 | 85.62 | 10.46 | 299 | 176 | 58.86 | 7.77 |

a: Neisseria meningitidis meningitis

b: The geometric mean concentration of serum antibody of group A

c: The geometric mean concentration of serum antibody of group C
