# Supplementary material for: Booster immunization of meningococcal meningitis vaccine among children in Hangzhou, China, 2014-2019
Source: PLoS One. 2021 May 25;16(5):e0251567. doi: 10.1371/journal.pone.0251567 (PMC8148366; doi:10.1371/journal.pone.0251567)
Supplement: S3 Table — Hangzhou’s financial per capita income from 2016 to 2018 shows that the urban per capita income is much higher than the rural per capita income. The disposable income of rural people is much lower than that of urban people. (DOCX) [file pone.0251567.s003.docx]

**S3 Table. Hangzhou's per capita income over the years from 2016 to 2018.** Hangzhou's financial per capita income from 2016 to 2018 shows that the urban per capita income is much higher than the rural per capita income. The disposable income of rural people is much lower than that of urban people.

| year | 2016 | 2017 | 2018 |
| --- | --- | --- | --- |
| Per capita GDP^a^ (ten thousand yuan) | 12.3 | 13.5 | 14.3 |
| Income of urban residents (yuan) | 52185 | 56276 | 61172 |
| Income of rural residents (yuan) | 27908 | 30397 | 33193 |

a: Gross domestic product
