# Supplementary material for: Booster immunization of meningococcal meningitis vaccine among children in Hangzhou, China, 2014-2019
Source: PLoS One. 2021 May 25;16(5):e0251567. doi: 10.1371/journal.pone.0251567 (PMC8148366; doi:10.1371/journal.pone.0251567)
Supplement: Attachment — Submitted filename: Response to Reviewers.docx [file pone.0251567.s004.docx]

**Response to the “Journal Requirements”:**

**1. Please ensure that your manuscript meets PLOS ONE's style requirements, including those for file naming. The PLOS ONE style templates can be found at……**

Authors’ response:

According to the requirements, we have checked the manuscript and ensure that our manuscript meets PLOS ONE's style requirements. If there are any formatting errors that we do not recognize, please specify them.

**2. Thank you for providing the date(s) when patient medical information was initially recorded. Please also include the date(s) on which your research team accessed the databases/records to obtain the retrospective data used in your study.**

Authors’ response:

As suggested, we have added the date(s) on which our research team accessed the databases/records used in our study in lines 102-103 as follows:” Our research team derived and analyzed the data from NIP in October 2020.”

**3. In your ethics statement in the Methods section and in the online submission form, please provide additional information about the data used in your retrospective study. Specifically, please confirm whether all data were fully anonymized before you accessed them.**

Authors’ response:

As suggested, we have added ethics statement in the Methods section and in lines 134-137 as follows:” Ethical considerations

This study was determined to be exempt from ethical review by the Hangzhou CDC institutional review board. The extraction of data from NIP and CNAEFIIS was safe, which was not linked to individual identiﬁers.”

**4. We note you have included a table to which you do not refer in the text of your manuscript. Please ensure that you refer to Table 1 in your text; if accepted, production will need this reference to link the reader to the Table.**

Authors’ response:

As suggested, we have linked the reader to the Table in line 143.

**Response to the reviewer's comments:**

**Response to the reviewer #1**

1. Typo: line 33 China Information Management System For Immunization Programming (CIMSFIP)

Authors’ response:

As suggested, we have corrected the word ”programming” in line 33.

Line 66 A large serogroup A and Serogroup C???

Authors’ response:

As suggested, we have rephrased sentence in line 66 as follows:” serogroup A or serogroup C.”

Line 74 and 79 two dots

Authors’ response:

As suggested, we have deleted one dot in line 74.

P value should be lower case in line 116

Authors’ response:

As suggested, we have changed “P value” into “*P* value” in line 128.

Typo in Table1) title is 2014-2018, should be 2014-2019

Authors’ response:

As suggested, we have changed “2014-2018” into “2014-2019” in line144.

Line 123 the number should be 1,376,919 and not 1376,919

Authors’ response:

As suggested, we have changed “1376,919” into “1,376,919” in line 140.

Use correct verb for the vaccine (not vaccined/ vaccinated is correct)

Authors’ response:

As suggested, we have checked all verb for the vaccine in lines 114,117,126,153,157,169,205.

Line 158 fourth dose is correct

Authors’ response:

As suggested, we have used the right “fourth” in line 178.

Line 200 We believe

Authors’ response:

We reedited the entire text of the paragraph where "we believed" is.

In lines 105-107 authors mentioned about household registration. Why not having more info about rural and urban vaccination distribution in the study? Which location have received more MPV-ACYW135 and why? Any idea about “more populous municipalities had lower AEFI reporting rate than less populous ones”?

Authors’ response:

As suggested, we have added analysis about rural and urban vaccination distribution in the study in lines 154-157,204-209. AEFI data cannot be summarized by region due to system upgrade, so this study did not analyze and discuss it.

Please describe why the MPV-ACYW135 is self-paid? What are the reasons that government does not support its free or subsidized use of MPV-ACYW135 vaccination?

Authors’ response:

As suggested, we have explained the reason “why the MPV-ACYW135 is self-paid” in line 81-85.

Any information about duplicate records and how did you handle it? Wu et al. (2019) found that “ an average of 3% duplicate records within provinces[China], and that duplicated record rates were higher in the eastern region than the western region”

Please check <https://doi.org/10.1016/j.vaccine.2019.08.070>

Authors’ response:

As suggested, we have checked “<https://doi.org/10.1016/j.vaccine.2019.08.070>” and explain how did we handle duplicate records in 119-122.

**Response to the reviewer #2**

1. Line 198 The authors claimed, “we believe that MPV-ACYW135 has advantages of simultaneously preventing a variety of diseases, reducing the number of injections and simplifying immunization procedures,” however, the authors did not provide any data such as serum antibody concentration in Hangzhou, cost-effective to demonstrate the MPV-ACYW135 better than MPV-AC. Statistical analysis also show there is no difference in the incidence of AEFI between MPV-AC and MPV-ACYW135. All data only show children in Hangzhou had a high coverage of MenV booster vaccination, the number of MPV-ACYW135 was used more frequently for children from 2014 to 2019. Why authors recommended to use MPV-ACYW135 instead of MPV-AC for free in Chinese children aged 3-6 years?

Authors’ response:

As suggested, we agree with Reviewer 2. We have revised our conclusions in lines 224-228 and made changes to the discussion section.

2. Discussion: line 186-195 cause controversy. For an example, “The AEFI data used in this paper was passively monitoring data, which may not truly reflect the occurrence of AEFI of MPV-AC or MPV195-ACYW135 to some extent.” Need to rewrite this paragraph.

Authors’ response:

As suggested, we have rephrased this paragraph in lines 216-223.

3. Minor error: line 127 from 2014 to 2018 in Hangzhou should be from 2014 to 2019 in Hangzhou.

Line 312 S3 Nma antibody levels May be S2 Nma antibody levels. (there no S3 table).

Authors’ response:

As suggested, we have changed “2014-2018” into “2014-2019” in line144 and “S2 Nma antibody levels”in line 326.
